# P-2156. Impact of Metagenomic Next-Generation Sequencing of Plasma Cell-Free DNA on Infectious Diseases Management in Adult and Pediatric Patients

**DOI:** 10.1093/ofid/ofae631.2310

**Published:** 2025-01-29

**Authors:** Olivia Tholt, Katie Lynn Hammer, Kiran Gajurel, Kevin S Buckley, Amanda Lefemine

**Affiliations:** Atrium Health, Charlotte, North Carolina; Atrium Health, Charlotte, North Carolina; Carolinas Medical Center, Atrium Health, Charlotte, North Carolina; Atrium Health Levine Children's Hospital/Wake Forest School of Medicine, Charlotte, North Carolina; Atrium Health, Charlotte, North Carolina

## Abstract

**Background:**

Plasma metagenomic next-generation sequencing (mNGS) is a noninvasive diagnostic test that uses cell-free DNA to identify a variety of pathogens. mNGS has the potential to identify difficult to detect pathogens and avoid invasive procedures while ensuring appropriate antibiotic use. However, the broad range of organisms detected makes it difficult to discern pathogens from colonization. The impact and ideal patient population for this test remain uncertain.**Figure 1.** Reason for Ordering mNGS

**Figure:**
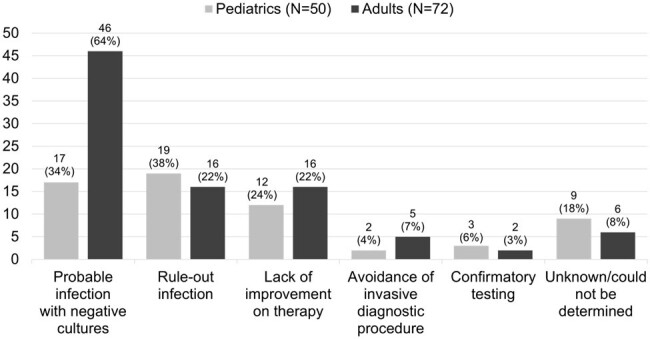
Note: patients could have more than one reason for ordering mNGS

**Methods:**

This was a retrospective evaluation of adult and pediatric patients who had mNGS ordered during their admission at Atrium Health's Carolinas Medical Center or Levine Children's Hospital from July 2019 through July 2023. The aim of this study was to characterize the use of mNGS including patient demographics, testing indication, result concordance with conventional testing and clinical impact of results. **Figure 2.** Suspected Site of Infection

**Figure:**
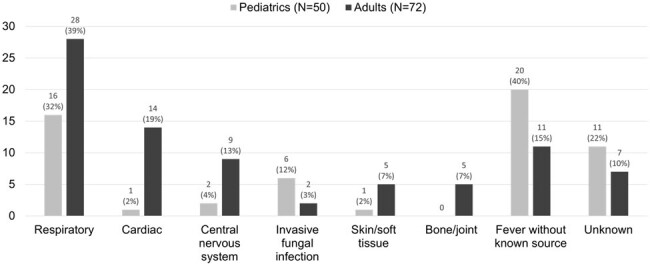
Note: patients could have more than one suspected site of infection

**Results:**

A total of 67 adult and 44 pediatric patients were included with 72 and 50 mNGS orders, respectively. Most of the patients were immunosuppressed including 72% of adult and 94% of pediatric patients. The most common reasons for ordering mNGS were probable infection with negative cultures, rule-out infection, and lack of improvement on current therapy (Figure 1). The suspected sites of infection are shown in Figure 2. There were 48 adult patients and 23 pediatric patients with a positive test. Of those that were positive, the results were fully concordant with conventional testing for 13% of adult and 26% of pediatric patients. At least one organism detected by mNGS was suspected to be the causative pathogen in 69% of adult and 43% of pediatric positive tests. Clinical impact was largely uncertain, and a negative impact was not identified in either group. The adult group had a positive impact in 33% of patients compared to 12% of pediatric patients. The most common positive impacts were confirmed and new diagnosis of which the most frequent suspected sites were cardiac and respiratory.

**Conclusion:**

mNGS was used primarily in immunocompromised patients with suspected, indeterminant infections. This data suggests mNGS may assist in the identification of organisms causing deep-seated infections or infections caused by pathogens otherwise difficult to detect but remains non-contributory to management in a majority of cases.

**Disclosures:**

All Authors: No reported disclosures

